# Using the “One Shot” Concept for Immediate Loading Implant Protocol in Edentulous Patient Rehabilitation with a Fixed Prosthesis: A 6-Year Follow-Up

**DOI:** 10.1155/2021/8872277

**Published:** 2021-02-24

**Authors:** Philippe Bousquet, Delphine Carayon, Jean-Cedric Durand

**Affiliations:** ^1^Department of Periodontology and Implantology, Faculty of Dentistry, University of Montpellier, Montpellier, France; ^2^Laboratoire Bioingenierie et Nanosciences UR-UM104, University of Montpellier, Montpellier, France; ^3^Department of Prosthetic Dentistry, Faculty of Dentistry, University of Montpellier, Montpellier, France

## Abstract

Immediate-loaded implants with a fixed prosthesis are a viable option for the restoration of edentulous ridges. Several procedures now allow for the fabrication of immediate-loading provisional and definitive prostheses. However, this complex treatment is not accessible to all patients with budget restrictions. By using a unique master model with a single titanium framework prosthesis can simplify and shorten the treatment, as well as reduce costs. After surgical placement of implant fixtures, an interim prosthesis was fabricated using a laser-welded definitive titanium framework. The prosthesis was fitted intraorally following the immediate loading protocols. The master cast model used to fabricate interim prosthesis was conserved and subsequently used in modifying the final prosthesis. After the healing process and complete soft tissue stability, an impression was made to register the clearance between the gingiva and resin. The light silicone material was directly injected under the prosthesis screwed in the mouth. In the master cast model, the stone was eliminated between the implants and a new plaster was poured to modify the crest profile with the posthealing new shape. With this modified model, it is possible to rehabilitate the denture to the new gingival anatomy in 3 to 4 hours and, if necessary, the tooth rearrangement. This “one shot” concept combines the single definitive titanium welded framework and limited laboratory work with a unique master model thereby decreasing the cost and the time of treatment.

## 1. Introduction

Complete edentulism is a serious public health concern that has not decreased over time despite advances in dentistry [1]. Rehabilitation with implant-supported total prosthesis offers a greater quality of life benefits than conventional total prostheses, the latter presenting with less functional stability [2]. To reduce processing time and patient discomfort, immediately loaded implants with a fixed prosthesis have been proposed [3, 4]. This technique has been well-documented rehabilitation of edentulous patients and has become a viable option due to its high survival rate [5–7]. In a short period of time, from several hours to 2 days [8, 9], a provisional restoration is made, screwed on the implants, and then used by the patient for the duration of the osseointegration period. It is very important that the temporary prosthesis avoids compressing the swollen soft tissues and not hamper the soft-tissue healing [10]. During the initial healing, a soft-tissue recession occurs. After osseointegration from 3 to 6 months, a complete-arch conventional impression registering the implant position and the surrounding tissue anatomy must be made to prepare the final master models for the definitive restoration. All these steps for the rehabilitation of edentulous patients increase the cost of treatment. A definitive metal-framework prosthesis directly positioned, twenty-four hours after implant placement, has been proposed to reduce the cost of treatment [11, 12]. This allows the exclusion of an interim prosthesis. Another technique can be the intraoral welding to manufacture the definitive restoration in a short time [13, 14]. However, at the end of complete healing reconstruction, after the implant surgery, the soft tissue level may potentially become an important space between the prosthesis and tissue [10]. The resulting consequences include the disturbance of speech, discomfort during or after mastication, and air escape between the prosthesis and tissue. In many cases, patients require new impressions and a new laboratory protocol for the fabrication of the final prosthesis, or at least for the relining of the restoration, all of which lead to additional expenses.

Such treatment is not readily accessible to all patients with budget restrictions. The simplified technique “one shot” concept described herein a clinical case and allows the transformation of a single prosthesis, which can be used as a temporary prosthesis with the criteria necessary for immediate loading, to a definitive prosthesis adapted to the tissue after healing.

## 2. Case Presentation

A 68-year-old man visited our clinic presenting with maxillary and mandibular complete dentures; the chief complaint was the instability of his mandibular denture. He did not smoke and showed no significant risk factors and no contraindications to surgery. Optional treatment was presented which included the two implant-supported overdentures and 4 to 6 dental implants with screw-retained fixed prosthesis. The patient agreed to be treated with an immediately loaded implant screw-retained fixed prosthesis and provided written informed consent.

### 2.1. Surgical and Prosthetic Protocols

Before surgery, impressions of the maxilla and mandible were made using irreversible hydrocolloid impression material (Alginoplast®, Kulzer Mitsui Chemicals Groups, Hanau, Germany) and diagnostic laboratory casts were poured with type III stones. The vertical dimension was established using facial reference marks, and the casts were mounted in a semiadaptable articulator (Artex® Amann Girrbach AG, Koblach, Austria) with a facebow transfer. The diagnostic setup for the final restoration was made. The mandibular tooth arrangement was duplicated in clear acrylic resin for the fabrication of a radiology guide. The number and position of implants were chosen on the computed tomography, in prosthetically favored positions, to reduce the cantilever length.

The preliminary step included implant surgery and an interim prosthesis. In this case, 6 implants were inserted with an insertion torque of more than 45 Ncm (Tapered ScrewVent®, Zimmer Biomet, Palm Beach Gardens, FL, USA). Abutments (tapered abutment TAC; Zimmer Biomet, Palm Beach Gardens, FL, USA) were tightened to 30 Ncm for the immediate loading protocol with a torque wrench.

**Figure 1:**
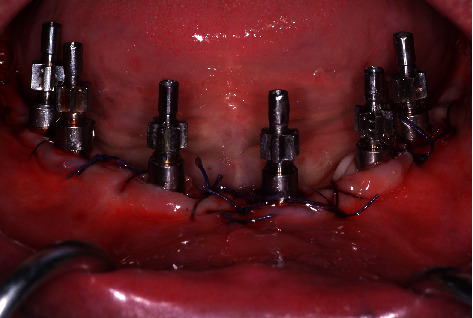
Impression transfer copings immediately after surgery.

Flap adaptation and suturing were performed. Analgesics were administered for pain control. Impression transfer copings (Tapered Abutment Direct Transfer, Zimmer Biomet, Palm Beach Gardens, FL, USA) were screwed on each implant abutment (Figure 1). A pickup open-tray impression was made with medium-bodied consistency polyether (Impregum™ Penta Soft, 3M Espe, Saint Paul, MN, USA) at the same time as the splitting impression transfer with resin (Luxabite®, DMG, Hamburg, Germany). After polymerization, the open tray was removed by loosening the transfer screws. Abutment analogs (Tapered Abutment Replica, Zimmer Biomet, Palm Beach Gardens, FL, USA) were tightened on impression transfer copings. The master cast was prepared and mounted on the articulator with the opposing cast using a bite record.

### 2.2. Laboratory Work

**Figure 2:**
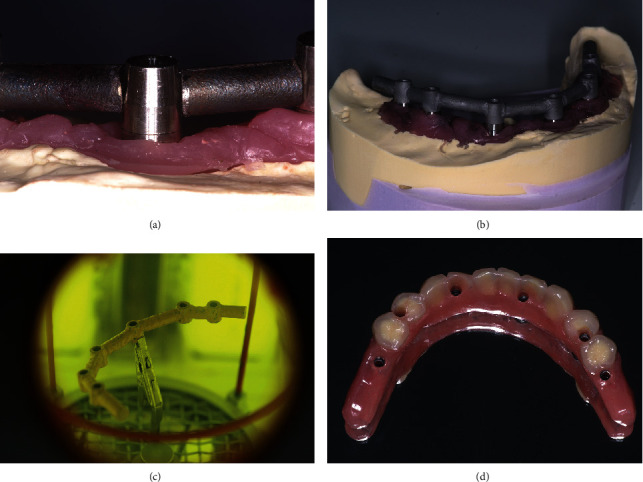
Definitive titanium implant framework fabricated using a laser-welding technique. (a) Segments of titanium rod adapted and inserted into the space between the titanium milled cylinders. (b) Definitive framework. (c) Pink opaquer layer was applied to increase prosthetic resistance under occlusal forces. (d) Interim prosthesis with acrylic teeth after finishing. Occlusion was shortened to the second premolar.

**Figure 3:**
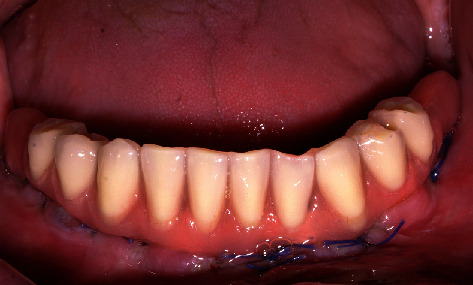
Interim prosthesis immediately after installation.

A definitive metal framework was obtained immediately after surgery, using titanium milled cylinders (Titanium Bar Coping 5 mm TTC5, Zimmer Biomet, Palm Beach Gardens, FL, USA) and segments of cylindrical 3.5 mm titanium rods, which were adapted and inserted between the cylinders screwed on the abutment replicas in the master cast (Figure 2(a)). The titanium implant framework was performed using laser welding, with two distal cantilever extensions (Figure 2(b)). For welded frameworks, a high-strength joint is required. The tungsten inert gas welding with efficient argon shielding is important to increase the cantilever resistance [15]. The passive fit of the framework was evaluated by radiographic examination and was clinically ensured using the screw test. The framework was sandblasted to improve the mechanical bond, and a pink opaquer layer was applied (Gingiva opaquer pink Nexco, Ivoclar Vivadent® AG, Schaan, Liechtenstein). Acrylic teeth were arranged in accordance with the setup, and the prosthesis was tried on for aesthetic evaluation and occlusion control. Relatively flat cusps were ensured for the occlusal pattern, contact on the cantilever was eliminated, and occlusion was shortened to the second premolar [16] (Figure 2(c)). This reinforced interim prosthesis was performed in less than 12 hours and delivered to the patient (Figure 3).

### 2.3. Posthealing Prosthetic Transformation

**Figure 4:**
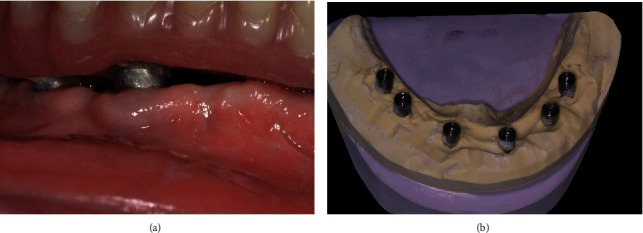
Preparation after osseointegration and gingival recession. (a) After healing, clearance between the alveolar crest and the denture. (b) Stone was eliminated between the implant abutment analogs on the master cast model.

**Figure 5:**
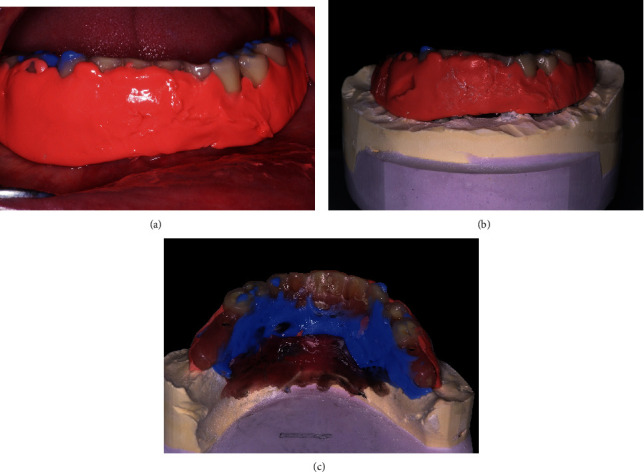
Posthealing prosthetic transformation. (a) Silicone impression material was injected under the prosthesis to fill the space, and occlusion was registered. (b) After the preparation of the model, the complex prosthesis and silicone are screwed onto the master cast model. (c) The restoration was boxed in the lingual area on the modified cast model.

Models on the articulator were preserved for the final transformation. After 3 months of osseointegration, the clearance between the gingiva and prosthesis was evaluated to edit the final prosthesis (Figure 4(a)). In the master cast model, the height of the stone between the implant analogs was reduced (Figure 4(b)). The interim restoration was unscrewed, and implants were checked with the prosthesis removed. To record the gingival recession and modify the denture in a short time, a tray adhesive for use with silicone (universal adhesive, Kulzer Mitsui Chemicals Groups, Hanau, Germany) was applied to the gingival portion of the restoration. The prosthesis was screwed back on the patient's implants. An impression was made with silicone light-bodied material directly injected under the prosthesis while it is firmly screwed intraorally to fill the space underneath. Silicone must cover the buccal and lingual parts of the prosthesis to isolate the potential undercuts (Figure 5(a)). Simultaneously, the occlusion was also recorded (Luxabite®, DMG, Hamburg, Germany).

**Figure 6:**
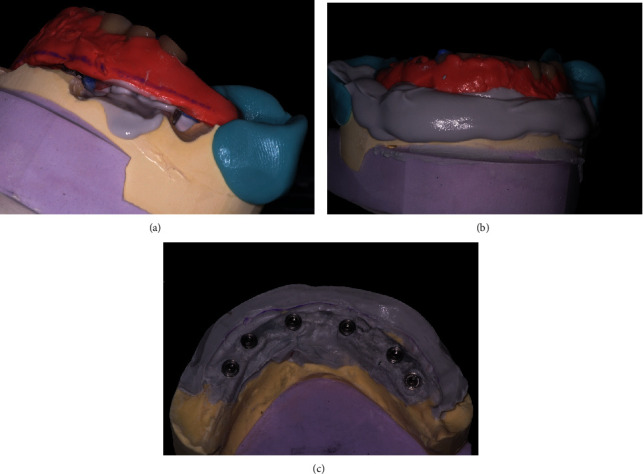
Master cast model modification. (a, b) New dental stone is poured into the space conserved on the buccal side. (c) Master cast model with the new crest anatomy.

The model was encased with wax and silicone, and a new plaster was poured into the space between the light silicone and the master cast model (reduced to modify the crest profile according to the new anatomy; Figures 5 and 6). The final prosthesis was adapted to the new crest, and first molars were added on the cantilevers. Regarding occlusion, in case of group guide or bibalanced occlusion, the acrylic teeth can be repositioned or replaced when necessary. Before the addition of chemopolymerizable resin (PERform Inkovac, Coltene/Whaledent AG, Altstätten, Switzerland), the denture was ground and the master model was prepared (wax-up and varnish). Pressure firing was conducted according to the manufacturer's recommendations. These laboratory steps were completed by grinding off excess resin and working on the shapes (embrasures and profiles) prior to polishing. In order to limit the risk of fracture or damage to the connection areas, a muffle technique was avoided in this case.

### 2.4. Results

**Figure 7:**
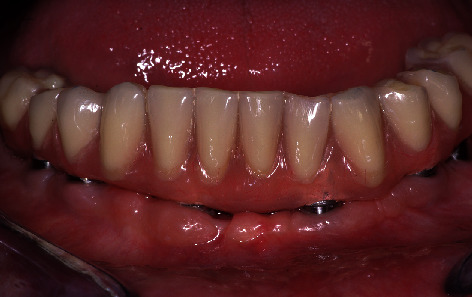
Final prosthesis. The molars and cantilevers were in occlusion, and a new adaptation to the crest was performed. The peri-implant tissues appeared healthy, indicating good oral hygiene level maintenance.

**Figure 8:**
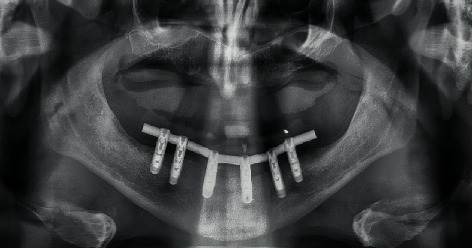
Radiographic panoramic control. After 6 years, bone level was stable.

A new prosthesis was delivered 4 hours after the impression. This work was conducted in a relatively short period of time and the treatment cost was lower. The function after 6 years was stable (Figures 7 and 8). After 3 years of function, prosthetic complications occurred due to fractures of the complete antagonist prosthesis. This problem was solved by reinforcement with a palatal metal frame.

## 3. Discussion

The use of a temporary prosthesis for full arch rehabilitation is important for the patient's comfort and to respect the immediate loading criteria. Conversion prosthesis [17] has been described as a technique for converting a transitional removable denture to a fixed provisional implant-supported prosthesis. This technique decreases the laboratory cost but increases the time of chair work. The precision of the prosthesis adaptation is often less than that of laboratory work. Moreover, it is difficult to incorporate metal renfort. Other authors have reported the feasibility of definitive all-acrylic resin prosthesis [18], but several studies have shown a better transmission of force and lack of micromovements on strengthened acrylic prosthesis [19, 20]. The micromovements of the implants during healing should not be more than 100 *μ*m [21].

The use of a definitive restoration, directly positioned after implant placement, in place of the provisional prosthesis, can be used to achieve the same work in a shorter period of time [11, 12]. However, in all these techniques, after osseointegration and complete healing of the soft tissue, space appears between the gingiva and prosthesis. Moreover, the tooth arrangement, particularly on the cantilever, and the occlusion require improvement.

In this “one shot” simplified technique, the definitive framework is immediately built on the master cast model, thereby conserving it. The passive fit of the interim prosthesis was controlled on the implants, which validates the master model. The use of laser welding on milled cylinders allows for, in time compatible with immediate loading, manufacturing a framework with the same precision fit than the CAD-CAM titanium framework and yields a more accurate fit than the frameworks produced with the lost-wax technique [22]. Moreover, the possibility of using a titanium framework decreases the corrosion and metal leaching in comparison to a chrome-cobalt alloy [23, 24].

For the posthealing prosthetic transformation, occlusion and vertical dimensions were established on the articulator during the first step, using a facebow. These parameters after interim prosthesis function can be changed, but in most cases in our experience, the modifications are not common. During the gingival recession, the occlusion is controlled and the master cast model can be remounted in the semiadaptable articulator if necessary. This technique can be used for relining prosthetics after a long period of use if excessive spaces appear troublesome, but it is important to conserve the master cast model. This work can also be done in a short time, and the treatment cost is lower.

This technique requires suitable equipment and experience in laser welding laboratory work [15]. This clinical case was free from complications during service; the only problem was with the complete antagonist denture, but this has been previously described in the literature [25]. Fracture of titanium framework at the posterior cantilever can occur and will require a new welding and localized refection of the resin. The most frequently observed problems are cosmetic, with teeth fracture or resin veneer fracture. The bonding between metal and composite is an important point for prosthetic resistance under occlusal forces.

## 4. Conclusion

From the patient's point of view, the financial aspect of implant treatment is important. “One shot” concept, which combines a definitive framework with recession impression after healing, allows for a single prosthesis, which can be used as a temporary prosthesis with the necessary criteria for immediate loading. Adaptation to the tissue after the healing phase is important for patient comfort and can be achieved in a short time. The definitive restoration meets the specifications of the implant-supported screw-retained total prosthesis. This prosthetic simplification reduces the cost and is accessible to a large number of patients. Further studies with larger samples are required to better evaluate this promising low-cost patient treatment option.
